# Human hypoblast formation is not dependent on FGF signalling

**DOI:** 10.1016/j.ydbio.2011.10.030

**Published:** 2012-01-15

**Authors:** Mila Roode, Kathryn Blair, Philip Snell, Kay Elder, Sally Marchant, Austin Smith, Jennifer Nichols

**Affiliations:** a Wellcome Trust Centre for Stem Cell Research, University of Cambridge, Tennis Court Road, Cambridge CB2 1QR, UK; b Department of Physiology, Development and Neuroscience, University of Cambridge, Tennis Court Road, Cambridge CB2 1QR, UK; c Department of Biochemistry, University of Cambridge, Tennis Court Road, Cambridge CB2 1QR, UK; d Bourn Hall Clinic, Bourn, Cambridge CB23 2TN, UK; e Centre for Reproductive Medicine, Barts and The London, West Smithfield, London EC1A 7BE, UK

**Keywords:** Pluripotency, Epiblast, Hypoblast, Fibroblast growth factor, Human ES cell derivation

## Abstract

Mouse embryos segregate three different lineages during preimplantation development: trophoblast, epiblast and hypoblast. These differentiation processes are associated with restricted expression of key transcription factors (Cdx2, Oct4, Nanog and Gata6). The mechanisms of segregation have been extensively studied in the mouse, but are not as well characterised in other species. In the human embryo, hypoblast differentiation has not previously been characterised. Here we demonstrate co-exclusive immunolocalisation of Nanog and Gata4 in human blastocysts, implying segregation of epiblast and hypoblast, as in rodent embryos. However, the formation of hypoblast in the human is apparently not dependent upon FGF signalling, in contrast to rodent embryos. Nonetheless, the persistence of Nanog-positive cells in embryos following treatment with FGF inhibitors is suggestive of a transient naïve pluripotent population in the human blastocyst, which may be similar to rodent epiblast and ES cells but is not sustained during conventional human ES cell derivation protocols.

## Highlights

► Segregation of epiblast and Gata4-positive hypoblast in human blastocysts ► Insensitivity of human hypoblast formation to FGF/Erk inhibition ► Persistence of Nanog-positive cells in human epiblast after FGF/Erk inhibition

## Introduction

The mouse embryo undergoes two crucial lineage segregation events before implantation in the uterus. The first is the separation of trophectoderm from the inner cell mass (ICM); the second is the segregation of the epiblast and hypoblast in the ICM. The trophectoderm and hypoblast lineages give rise to extra-embryonic tissues that facilitate the implantation of the embryo into the uterus and formation of the placenta. The epiblast forms a pool of pluripotent cells that will give rise to the foetus in collaboration with patterning signals from the extra-embryonic tissues (Beddington and Robertson, 1999; Gardner and Beddington, 1988). The pre-implantation epiblast is also the source of embryonic stem (ES) cells (Brook and Gardner, 1997).

The separation of the trophectoderm and ICM in mouse embryos is marked by mutually exclusive expression of the two transcription factors Oct4 and Cdx2 (Niwa et al., 2005). The segregation of epiblast and hypoblast from the cells of the ICM is suggested to involve Nanog and Gata6. These two factors are initially expressed in an overlapping manner in the ICM but then become mutually exclusive in the late mouse blastocyst (Chazaud et al., 2006; Plusa et al., 2008). Epiblast and hypoblast lineages are morphologically distinct within the murine ICM by embryonic day (E) 4.5, just before implantation.

Studying the mechanisms of early lineage segregation has provided insight into how the pluripotent epiblast is specified, and this has enabled development of efficient strategies to isolate ES cells from murine embryos, either by physical separation (Brook and Gardner, 1997) or application of specific inhibitors to block differentiation (Buehr et al., 2008; Li et al., 2008; Nichols et al., 2009; Ying et al., 2008).

Pluripotency is defined as the ability of a cell to give rise to different tissues representative of all three of the embryonic germ layers: ectoderm, mesoderm and endoderm. It can be considered as two states (Nichols and Smith, 2009). Naïve (or ‘ground state’) pluripotency is represented by the newly segregated pre-implantation epiblast and rodent ES cells. Following implantation, the epiblast responds to signals from the extra-embryonic tissues and becomes primed for differentiation. Primed pluripotency is also exhibited by the epiblast stem cell lines (EpiSCs) derived from post-implantation epiblasts in culture (Brons et al., 2007; Tesar et al., 2007). In addition to their ability to give rise to multiple tissue types, naïve and primed pluripotent cells are both characterised by expression of the core pluripotency factors Oct4, Sox2 and Nanog, but differ in their expression spectrum of other genes, their culture requirements and their ability to resume normal development when placed in the embryonic environment. Interestingly, although pluripotent cell lines have been derived from primate blastocysts (Suemori et al., 2001; Thomson et al., 1995; Thomson et al., 1998), these cells more closely resemble EpiSCs than murine ES cells (Brons et al., 2007; Tesar et al., 2007). Significantly, both primate ES cells and EpiSCs require exogenous FGF in order to self-renew, in contrast to rodent ES cells.

Naïve pluripotency can readily be captured from embryos of mice and rats when fibroblast growth factor (FGF)/Erk signalling are inhibited in combination with glycogen synthase kinase 3 (GSK3) inhibition, a system known as ‘2i’, short for ‘two inhibitors’ (Buehr et al., 2008; Li et al., 2008; Nichols et al., 2009; Ying et al., 2008). Analysis of murine embryos cultured from the 8-cell-stage in 2i revealed that the resulting ICM consisted almost entirely of cells expressing high levels of Nanog and no Gata4, indicating that putative hypoblast cells had become epiblast (Nichols et al., 2009). The efficient derivation of murine ES cells in 2i may therefore be attributable in part to blockade of hypoblast. Further evidence that formation of hypoblast in the mouse is dependent upon FGF signalling is provided by the demonstration that supplementation of the embryo culture medium with high levels of recombinant FGF4 diverts ICM cells to hypoblast (Yamanaka et al., 2010).

The 2i culture regime provides a highly selective environment in which cells not exhibiting properties of naive pluripotency fail to thrive, and are thus eliminated. EpiSCs and human ES cells do not survive in 2i (Guo et al., 2009; Nichols and Smith, 2009). So far there have been no reports of the derivation of naïve pluripotent cells from non-rodent embryos. For practical purposes, naïve human ES cells would be highly desirable, but attempts to generate human ES cells by culturing embryos in 2i have failed (JN, MR, unpublished). We have attempted to find an explanation for this failure by first investigating lineage segregation in human embryos, then analysing their responses to the application of inhibitors, specifically in the maintenance of Nanog expression and the emergence of Gata4-expressing cells.

## Materials and methods

### Human embryos

Human embryos surplus to IVF requirements were donated to research after informed patient consent, with approval of local ethics committees and the UK Human Fertilisation and Embryology Authority (Research licence R0178). Embryos were obtained from Bourn Hall Clinic, Cambridgeshire. Embryos were thawed using EmbroThawTM Kit (EMF40_T, Fertipro) according to the manufacturer's instructions. Embryos were cultured to day 3 in EmbryoAssist™ (12140010, Origio), after which they were changed to BlastAssist® (12160010, Origio) until the appearance of a cavity, upon which they were transferred to N2B27 medium (SCS-SF-NB-02, Stem Cell Sciences) (Nichols and Ying, 2006; Ying and Smith, 2003) for further culture. Embryos were cultured in 50 μl drops of medium under mineral oil (MINOIL500, Fertipro) and changed to fresh medium every 2 days. Embryos were cultured in a humidified atmosphere at 5% oxygen, 7% carbon dioxide and 37 °C. Embryos were exposed to inhibitors from day 3 of development.

Culture medium was supplemented with inhibitors in the combinations specified in the text: PD0325901, a Mek inhibitor with an IC_50_ value in the 20–50 nM range in human cells (Ciuffreda et al., 2009), 0.5 μM or 1 μM; Chir99021, a GSK3β inhibitor with an IC_50_ value of 10 nM in human cells (Ring et al., 2003), 3 μM (both synthesised in the Division of Signal Transduction Therapy, University of Dundee, Dundee, UK) and PD173074 (Sigma), a pan-FGF receptor inhibitor with an IC_50_ value in the range of 5–22 nM for human FGFR1–5 (Byron et al., 2008; Grand et al., 2004; Mohammadi et al., 1998; Skaper et al., 2000; St Bernard et al., 2005; Stavridis et al., 2007), 100 nM.

### Rodent embryos

The strains of mice used in this study were MF1 outbred, and F1 hybrids between C57BL/6/Ola and CBA/Ca. The strain of rat used was Sprague–Dawley (SD). Animals were maintained by in-house breeding on a lighting regime of 14 hours light and 10 hours darkness with food and water supplied ad libitum. Prior to caging with stud males, female mice were oestrous selected by visual inspection of the vagina (Champlin et al., 1973). Female rats were selected for oestrus using a vaginal impedance monitor (Muromachi Kikai, Tokyo, Japan, MK-11)(Koto et al., 1987). Detection of a copulation plug the following morning was used to confirm successful mating; the resulting embryos were then considered to be E0.5. For all experiments, the embryos from at least two females were pooled and randomly assigned to experimental groups. Mouse and rat embryos were flushed from oviducts using M2 medium at E2.5 and E3.5 respectively. Mouse embryos were cultured in BlastAssist® for 2 days before being moved to N2B27 as expanded blastocysts. Rat embryos were cultured in BlastAssist® for 1 day, after which they were switched to N2B27. Embryos were cultured at 5% oxygen, 7% carbon dioxide and 37 °C.

### Immunostaining

Embryos were processed for immunohistochemistry as described previously (Nichols et al., 2009). Briefly, they were fixed in 4% paraformaldehyde in PBS for 15 minutes, then rinsed in PBS containing 3 mg/ml polyvinylpyrolidone (PBS/PVP; P0930, Sigma), permeabilised in PBS/PVP containing 0.25% Triton X-100 (23,472-9, Sigma) for 30 minutes and blocked in blocking buffer, which comprised PBS containing 0.1% BSA, 0.01% Tween 20 (P1379, Sigma) and 2% donkey serum. Primary antibodies were Oct4 (C-10 sc-5279, Santa Cruz Biotech, Santa Cruz, CA, USA), Nanog (ab21624 or ab21603, Abcam, Cambridge, UK), Gata4 (C-20 sc-1237, Santa Cruz), Gata6 (R&D Systems, AF1700) and Sox17 (R&D Systems, AF1924). Antibodies were diluted 1:100 (Oct-4 was used at 1:200) in blocking buffer and embryos were incubated in the appropriate antibody solution at 4 °C overnight. They were rinsed three times in blocking buffer for 15 minutes each, and incubated in secondary antibody solution for 1 hour at room temperature. Secondary antibodies raised in Donkey labelled with Alexa fluorophores (Invitrogen, Paisley, UK) were diluted 1:500 in blocking buffer (anti-mouse IgG_2b_ for Oct4:647; anti-rabbit IgG for Nanog:488; anti-goat IgG for Gata4, Gata6 and Sox17:555). Embryos were then rinsed three times in blocking buffer, incubated briefly in increasing concentrations of Vectashield (H-1200, Vector Labs, Peterborough, UK) before mounting on glass slides in small drops of concentrated Vectashield with DAPI, and subsequently sealed with nail varnish.

### Image collection and analysis

Embryos were imaged on either a Leica TCS SP5 confocal microscope or Revolution XD Confocal System (Andor). Reconstructions of three-dimensional images from sections and cell counts were performed using Leica or Imaris software.

### Statistical analysis

Probability (P) values were established using Student's *t*-test for comparison between two samples.

## Results

### Human embryo culture to the late blastocyst stage

Human embryos were previously thawed and cultured in standard IVF medium to the blastocyst stage (day 6–7) at 20% oxygen. Blastocyst formation rates were low (17.5%, 25/143) and many blastocysts did not progress beyond the initial cavitation stages. The use of 5% oxygen has been reported to be beneficial for blastocyst formation rates (Dumoulin et al., 1999; Waldenstrom et al., 2009). By reducing the concentration of oxygen in the culture regime to 5%, our blastocyst formation rates increased significantly (35.1%, 67/191), with many embryos progressing to day 7 and older. Therefore, for all subsequent experiments a humidified atmosphere of 5% oxygen, 7% carbon dioxide and 37 °C was adopted.

### Putative hypoblast is segregated by day 7 of human development

The interplay between Nanog and Gata6 is suggested to be crucial for the epiblast/hypoblast fate decision in mouse embryos (Plusa et al., 2008). Although Nanog protein has been shown to localise to a sub-population of cells in the ICM of human embryos at the mid/late blastocyst stage (Cauffman et al., 2009; Hyslop et al., 2005; Kimber et al., 2008), the identity of the Nanog-negative ICM cells has not yet been investigated. We therefore investigated the expression of the hypoblast markers Gata6, Gata4 and Sox17 in human blastocysts.

**Fig. 1:**
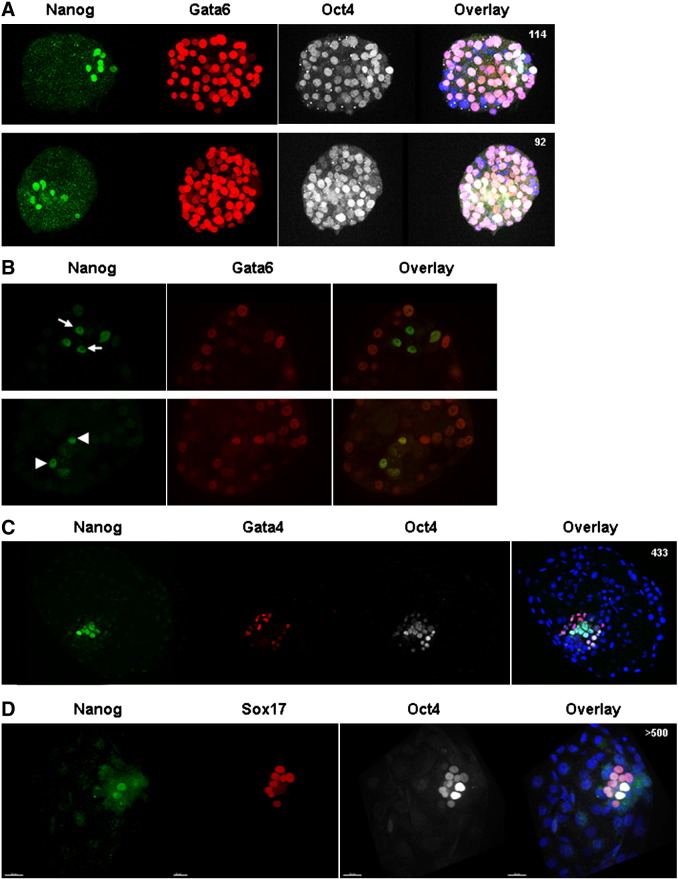
Human embryos segregate putative hypoblast by day 7 of development. Human embryos were thawed and cultured in standard IVF medium until they formed cavitated blastocysts, upon which they were moved to N2B27 medium. Embryos were fixed and immunostained for Oct4 (white), Nanog (green) and Gata6 (red). At day 6 of *in vitro* development (A) Nanog is restricted to a few cells within the embryo, whilst Gata6 and Oct4 are broadly expressed. Confocal images of two representative embryos with a maximum projection of the 3D reconstruction of the blastocyst are shown. (B) A single slice in a z stack of each of the two embryos shown in (A), indicating that Nanog and Gata6 can both be expressed highly in the same cell (arrowheads) or that Gata6 can be low as Nanog is high (arrows). (C and D) Embryos were developed to day 7 *in vitro* and immunostained for Nanog (green), Oct4 (white) and Gata4 (red) (C) or Sox17 (red) (D). In contrast to the stainings observed at day 6, Oct4 is restricted to the cells of the ICM. Gata4 and Sox17 are restricted to a subset of cells within the embryo, distinct from the Nanog positive cells: the putative hypoblast. In all embryos nuclei were counterstained with DAPI (blue). The total number of cells in each embryo is written in the top right hand corner of the panel.

We first examined human embryos at 6 days post-fertilisation. Nanog is confined to a few inner cells within the embryo (Fig. 1A). Gata6 and Oct4 are widely expressed throughout the embryo, with levels varying between individual cells, consistent with previous data (Cauffman et al., 2005; Cauffman et al., 2006; Chen et al., 2009; Kimber et al., 2008). Some inner cells exhibit high levels of Nanog and low levels of Gata6 (Fig. 1B, arrows), whilst in other cells both markers are expressed at about the same level (Fig. 1B, arrowheads). This pattern is similar to that observed in the early mouse blastocyst (Plusa et al., 2008).

**Fig. 2:**
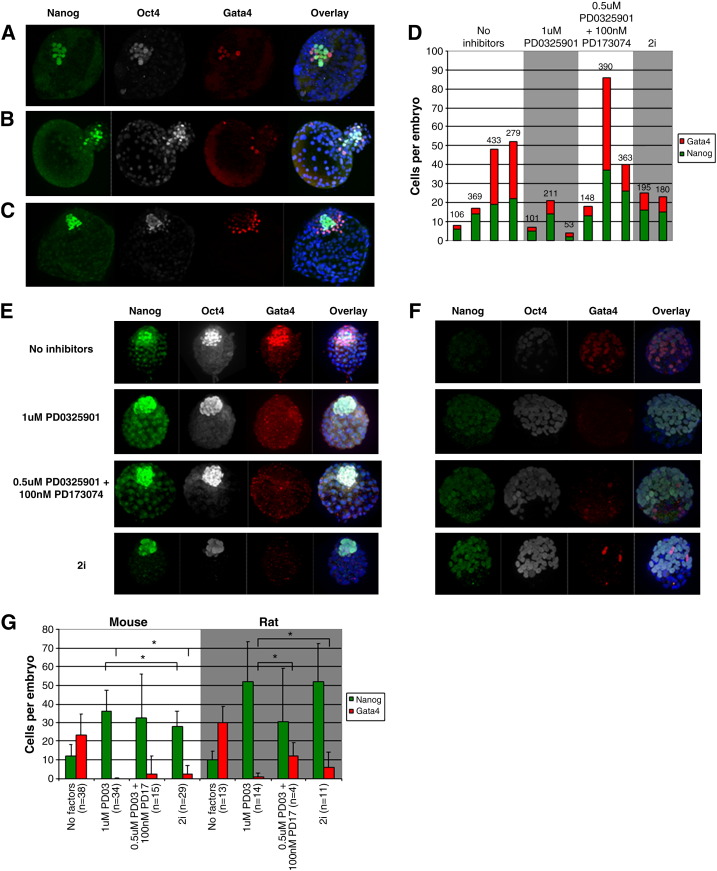
Effect of FGF/Erk signalling inhibition on human epiblast and hypoblast compared with mouse and rat. Human embryos were thawed and cultured in standard IVF medium until they formed cavitated blastocysts, upon which they were moved to N2B27 medium. Embryos were exposed to inhibitors from the 6–8 cell stage and developed until day 7 *in vitro*. Embryos were immunostained for Oct4 (white), Nanog (green) and Gata4 (red). Confocal images were taken and 3D reconstructions of the embryos created. The addition of 1 μM PD0325901 (A), 2i (B) or 0.5 μM PD0325901 and 100 nM PD173074 (C) did not eliminate the segregation of the putative hypoblast as indicated by the expression of Gata4. (D) Blastocysts were variable in their number of Nanog and Gata4-expressing cells within each experimental group and across experimental groups. This may be due to the inherent variation between human embryos *in vitro*. The number of cells per embryo is written above each bar in the graph. Mouse (E) and rat (F) embryos were cultured from the 8-cell-stage under the same culture regime as human embryos. The addition of small molecules that inhibit the FGF/Erk pathway result in the loss of hypoblast in these embryos, indicating that hypoblast formation is dependent on FGF signalling in both the mouse and rat. The Nanog antibody utilised has a lower affinity for the rat protein (F) than the mouse (E). (G) Cells of the epiblast and hypoblast were counted in 3D reconstruction of embryos. * indicates a *P* < 0.05 indicating a statistically significant difference between two data sets. The statistical differences of the no factors group and inhibitor conditions were not plotted for clarity. In all embryos nuclei were counterstained with DAPI (blue).

At day 7 of development, Gata4 and Sox17, both markers of differentiated hypoblast, are restricted to a narrow subset of cells within the embryo (Fig. 1C). Significantly, at this stage of development, this pattern is mutually exclusive with Nanog expression. Some embryos show Gata4 or Sox17-positive cells in what appears to be an epithelial layer overlying the Nanog-positive epiblast on the blastocoelic surface (Figs. 1C and 2C). This mirrors delamination of the hypoblast seen in rodent blastocysts. Oct4 protein is much more restricted to cells of the ICM than in earlier blastocysts, with staining in both the epiblast and hypoblast (Fig. 1C), as is the case with early murine hypoblast (Silva et al., 2009). This may be due to slight differences in the developmental age of the embryos, most likely reflecting variability in their development in vitro. These embryos tended to exhibit reduced total cell number, consistent with this (Fig. 1C, D). These observations suggest that the human embryo at day 7 resembles the mouse embryo at E4.5 when all three embryonic lineages can be distinguished.

### Hypoblast segregation is not dependent upon FGF/Erk signalling

FGF/Mek inhibition in mouse preimplantation embryos has a striking effect on lineage segregation (Chazaud et al., 2006; Nichols et al., 2009; Yamanaka et al., 2010). Inhibition of FGF signalling diverts all cells of the ICM to the epiblast fate, bypassing the hypoblast. We were interested in investigating whether the formation of human hypoblast is also dependent on FGF signalling. If so, this would indicate that the segregation of these lineages may be based on a conserved mechanism. We performed experiments with human embryos, adding inhibitors from the 6–8 cell stage. 1 μM PD0325901 is effective to block the formation of hypoblast in murine embryos if applied before its segregation (Nichols et al., 2009). However, all human embryos treated with 1 μM PD0325901 that developed to late blastocysts showed Gata4 expression in a subset of cells, separate from the Nanog-expressing population (Fig. 2A, D). To test whether an alternative downstream pathway for FGF signalling is adopted during human hypoblast induction, we introduced an inhibitor of the FGF receptor, PD173074, in combination with PD0325901. Using this combination, Gata4-positive cells were still identified in all the human embryos investigated (Fig. 2C, D). We examined whether PD0325901 could work synergistically with Chir99021, since these are the components of 2i that effectively maintain naïve pluripotency in cultures of murine ES cells (Ying et al., 2008). Gata4-positive cells were still observed in embryos developed under these conditions (Fig. 2B, D). In all three conditions that target FGF/Erk signalling, human embryos possess Gata4 and Nanog-positive cells that are exclusive to one another. Thus, the segregation of epiblast and hypoblast within the human embryo appears not to be dependent on FGF signalling.

To eliminate the human-specific culture regime as a potential variable in the response of embryos to FGF signalling inhibition, we cultured murine embryos from the 8-cell-stage under identical conditions. Mouse embryos responded as previously reported to 1 μM PD0325901 and 2i (Nichols et al., 2009) (Fig. 2E, G). We also used the combination of PD0325901 and PD173074 that has been shown to block hypoblast formation (Yamanaka et al., 2010). This also prevented formation of Gata4 positive cells. Finally, we cultured rat embryos under these conditions and found very similar responses (Fig. 2F, G). This indicates that the effect of FGF inhibition is not specific to the mouse.

In both mouse and rat embryos, 1 μM PD0325901 reproducibly blocks the formation of the hypoblast. The addition of Chir99021 to PD0325901 (2i) seems to facilitate some hypoblast cells escaping this block, especially in the rat embryo (Fig. 2G). The combination of 0.5 μM PD0325901 and PD173074 reduces the emergence of hypoblast compared to control embryos, but is not as effective in blocking hypoblast as 1 μM PD0325901. Despite the consistency of 1 μM PD0325901 in its effects on mouse and rat embryos, it did not eliminate the formation of Gata4-positive cells in the human embryo. However, it should be noted that culturing human embryos for extended periods in FGF signalling inhibitors resulted in no detectable detrimental effect on the Nanog positive population of cells.

## Discussion

The first two lineage decisions that mouse embryos undertake during preimplantation development have been extensively studied. Detailed examination of these decisions has not been performed in human embryos due to the scarcity of available material and the sub-optimal nature of culture regimes. Whilst the differentiation of ICM and trophectoderm has been observed in human embryos (Cauffman et al., 2009; Chen et al., 2009), there has been little evidence for the segregation of the ICM into epiblast and hypoblast. Nanog has previously been shown to be restricted to a subset of ICM cells within the embryo at the blastocyst stage (Cauffman et al., 2009), but it was not known if the Nanog-negative cells within the ICM represented hypoblast. Fig. 1C demonstrates Gata4 expression in cells negative for Nanog, indicating that the hypoblast is segregated from the epiblast within the ICM by day 7 of human development. A population of cells negative for Nanog is also immunoreactive to Sox17 (Fig. 1D), an additional marker of hypoblast in the mouse, providing further evidence that hypoblast is formed in human embryos.

We have previously reported that the segregation of hypoblast in mouse embryos is dependent upon FGF signalling (Nichols et al., 2009). We sought to examine if this was also the case in human embryos. Mouse embryos behaved as we have previously reported when exposed to small molecule inhibitors targeting the FGF pathway when we cultured them in the human embryo culturing regime (Fig. 2E, G). The effect of inhibiting FGF signalling on lineage allocation in mouse embryos is not specific to this species: rat embryos behaved in a similar way (Fig. 2F, G). This observation indicates that the role of FGF/Erk signalling is conserved amongst the early embryos of these two species.

The effect of FGF inhibition on human embryos shows a number of differences from the effects observed in rodents, even though the concentrations used were well above their respective IC_50_ values. Gata4-positive hypoblast still forms under conditions where it is blocked in rodent embryos (Fig. 2A–D), indicating that the formation of human hypoblast is not dependent on FGF signalling. The molecular mechanism of hypoblast segregation in the human embryo is still unknown. Possible candidates include the transforming growth factor (TGF) β family proteins that operate via SMAD transcription factors. Human ES cell lines can be propagated in a state of primed pluripotency in the presence of Activin (Vallier et al., 2005), a member of this family. Furthermore, TGFβ has been shown to maintain self-renewal of trophectoderm stem cells (Erlebacher et al., 2004). It may therefore be anticipated that blocking this pathway could shield pluripotent cells from diverting to extra-embryonic lineages. The mitogen-activated protein kinase P38α has been suggested to play a role in branching morphogenesis of embryonic lung epithelium (Liu et al., 2008), a derivative of the definitive endoderm that shares an overlapping molecular profile with the hypoblast. Although we have failed to detect any effect of suppression of hypoblast formation by applying inhibitors of either of these pathways to early mouse or rat embryos (data not shown), they have not yet been applied to human embryos.

The Nanog-positive epiblast compartment is not reduced in human embryos when they are cultured in the presence of inhibitors for FGF/Erk and Gsk3 signalling (Fig. 2D). Human ES cells cannot survive exposure to 2i (Nichols and Smith, 2009). If the Nanog-positive cells in the human embryo were the in vivo counterpart of human ES cells, they may be expected to deteriorate in the presence of small molecules targeting FGF/Erk signalling. The survival of Nanog-positive cells within the embryo suggests that it may ultimately be possible to isolate cells that are independent of FGF/Erk signalling from these embryos (Hanna et al., 2010; Smith, 2010).
